# Supplementary material for: Gaussian hierarchical latent Dirichlet allocation: Bringing polysemy back
Source: PLoS One. 2023 Jul 12;18(7):e0288274. doi: 10.1371/journal.pone.0288274 (PMC10337977; doi:10.1371/journal.pone.0288274)
Supplement: S1 File — (PDF) [file pone.0288274.s001.pdf]

Supporting information for Gaussian Hierarchical Latent Dirichlet Allocation: Bringing Polysemy Back

Takahiro Yoshida,[1] Ryohei Hisano,[2] Takaaki Ohnishi[3]
[1]The Canon Institute for Global Studies, Tokyo, Japan
[2]Graduate School of Information Science and Technology, Tokyo, Japan
[3]Graduate School of Artificial Intelligence and Science, Rikkyo University, Tokyo, Japan

More Information on the Datasets used in the Paper

Here we describe how we collected the datasets. For all the data, we confirm that the collection of this data complies with the terms of service.

The Wikipedia dataset was created from DBpedia-2016 long abstract data (?) which is an open dataset. Each long abstract in the DBpedia dataset has several attached labels to classify each article. We focused on the following six categories: “Rivers,” “Banks/Financial,” “Military,” “Law,” “Mathematical,” and “Football.” We sampled evenly from these categories to create a corpus of 6,000. The main feature of this dataset is the inclusion of the “Rivers” and “Banks/Financial” categories. By randomly sampling from these categories, we created a corpus that used “bank” both a financial institution and a steep place near a river.

Amazon review data is available at http://jmcauley.ucsd.edu/data/amazon/ (?) which is an open data set. We sampled evenly from the following five categories: “Electronics,” “Video Games,” “Home and Kitchen,” “Sports and Outdoors,” and “Movies and TV,” and created a corpus of 6,000.

Reuters data is a news dataset web-scraped from Reuters news. We collected 6,000 news stories from the period Jan 2016 to Feb 2016. This last dataset could not be made public to

comply with the term of service. The data could be bought from Refinitiv's Machine Readable News service.
